# Barriers to and Facilitators of User Engagement With Digital Mental Health Interventions: Systematic Review

**DOI:** 10.2196/24387

**Published:** 2021-03-24

**Authors:** Judith Borghouts, Elizabeth Eikey, Gloria Mark, Cinthia De Leon, Stephen M Schueller, Margaret Schneider, Nicole Stadnick, Kai Zheng, Dana Mukamel, Dara H Sorkin

**Affiliations:** 1 University of California Irvine Irvine, CA United States; 2 University of California San Diego San Diego, CA United States

**Keywords:** mHealth, eHealth, mental health, depression, anxiety, behavior, mobile phone

## Abstract

**Background:**

Digital mental health interventions (DMHIs), which deliver mental health support via technologies such as mobile apps, can increase access to mental health support, and many studies have demonstrated their effectiveness in improving symptoms. However, user engagement varies, with regard to a user’s uptake and sustained interactions with these interventions.

**Objective:**

This systematic review aims to identify common barriers and facilitators that influence user engagement with DMHIs.

**Methods:**

A systematic search was conducted in the SCOPUS, PubMed, PsycINFO, Web of Science, and Cochrane Library databases. Empirical studies that report qualitative and/or quantitative data were included.

**Results:**

A total of 208 articles met the inclusion criteria. The included articles used a variety of methodologies, including interviews, surveys, focus groups, workshops, field studies, and analysis of user reviews. Factors extracted for coding were related to the end user, the program or content offered by the intervention, and the technology and implementation environment. Common barriers included severe mental health issues that hampered engagement, technical issues, and a lack of personalization. Common facilitators were social connectedness facilitated by the intervention, increased insight into health, and a feeling of being in control of one’s own health.

**Conclusions:**

Although previous research suggests that DMHIs can be useful in supporting mental health, contextual factors are important determinants of whether users actually engage with these interventions. The factors identified in this review can provide guidance when evaluating DMHIs to help explain and understand user engagement and can inform the design and development of new digital interventions.

## Introduction

### Background

Nearly 1 in 5 adults in the United States experience a mental illness at some moment in their life [1]. Yet, accessing treatment for mental health problems can be difficult. Common barriers to mental health care include stigma, lack of available and evidence-based services, and inability to afford services [2,3]. In addition, people not diagnosed with a mental illness can experience periods of poor mental health and may benefit from support, although they have not sought professional treatment with a mental health provider. For instance, 73% of people surveyed in the United States experience stress related to money, work, and family responsibilities at a level that affects their mental health [4]. The translation of psychosocial interventions into digital formats, deemed digital mental health interventions (DMHIs), has the potential to overcome some existing barriers to traditional care and increase access to mental health support and resources.

DMHIs can be delivered via smartphone apps, internet websites, wearable devices, virtual reality, or video games [5] and range from self-guided DMHIs to those integrated with human support or traditional therapy [6]. Although some DMHIs have been shown to be as effective as traditional mental health services (eg, psychotherapy and pharmacotherapy) in improving mental health conditions such as depression [7] and can lead to greater reductions in anxiety compared with usual care [8], engagement with these technologies remains to be an ongoing issue, varies from study to study, and is typically lower in real-world use than research studies [9]. For example, a review in 2018 found that participant adherence to internet-delivered cognitive behavioral therapy (CBT) can range from 6% to 100% [10]. Similarly, systematic comparisons in 2018 and 2019 on self-help DMHIs found that real-world uptake varies widely [9,11], and acceptability can be lower than traditional treatment [12].

This paper aims to systematically review the literature on DMHIs to identify common barriers and facilitators that may influence user engagement with these interventions. There are different ways to define user engagement. For example, engagement can be referred to as the time a user spends on an intervention. However, the time spent on an intervention varies between different types of interventions, and little time spent using a DMHI does not have to be a negative feature per se. To get a comprehensive understanding of people’s use of DMHIs, we use a broader definition of user engagement. In this review, *user engagement* refers to a user’s uptake and sustained interactions with a digital intervention, which includes interest in adopting an intervention as demonstrated by signing up for the digital intervention, initial uptake as demonstrated by engaging with features of the digital intervention as part of the study, at a minimum during a demonstration as part of the study, and continued use of an intervention.

### Understanding User Engagement With DMHIs

A range of factors can influence engagement with DMHIs, such as the relevance of information to the user provided by a digital intervention [13], a lack of user motivation to persist with a self-guided intervention [14], and poor user experience with the technology [15]. Although previous studies have each reported on *some* factors that can influence engagement, given a particular technology or context, a review is lacking that brings all these findings together. It is important to investigate the multitude of factors to fully understand the reasons for high versus low engagement. Previous reviews have highlighted the variability in engagement and uptake, analyzing both DMHIs published in the academic literature [9-11] and publicly available mental health apps in app stores [16]. However, these analyses did not report on factors related to this variability in engagement. This review seeks to address this gap by identifying the most common overarching factors that affect engagement.

Although analyzing engagement metrics of commercial apps can be used to examine variability in engagement, user studies are valuable to understand the underlying reasons why people may engage with some interventions more than others. For the purpose of this review, we focus on reviewing the academic literature.

Researchers and developers of DMHIs can use this knowledge to inform evaluations of engagement and the development of new digital interventions. In addition, it may provide insights into what services and facilitating conditions need to surround DMHIs to promote technology-enabled services and may help mental health service providers in selecting suitable interventions for their clients.

This review focuses on common mental health issues, such as depression, anxiety, psychological well-being and distress, and stress. There may be different barriers or facilitators for user engagement with other specific, serious mental illness interventions (eg, psychosis intervention) that are beyond the scope of this paper.

## Methods

### Inclusion Criteria

The inclusion and exclusion criteria of articles for this review are presented in Textboxes 1 and 2, respectively.

**Inclusion criteria.**

- Report on an intervention aimed to improve mental health, psychological well-being, anxiety, depression, stress, and/or mood
- Report on an intervention delivered in a digital format, such as a smartphone app or website
- Report on some aspects of user experience (eg, usability, user satisfaction, and user feedback)
- Report on factors that affected user experience
- Include participants aged ≥16 years (eg, child and adolescent samples were excluded)
- Report on an empirical study (eg, literature reviews that synthesized findings from other articles, columns, opinion pieces, comments or replies, and editorials were excluded)
- Be a peer-reviewed article (eg, dissertations were excluded)
- Be written in English

**Exclusion criteria.**

- Report on interventions that have a mental health component but do not have mental health as a primary intervention target (eg, an app that is primarily focused on physical pain symptoms, with a mental health component)
- Report on interventions that only serve as an appointment booking system for in-person therapy
- Report on interventions that are used as a component during an in-person session but cannot be used remotely outside of these sessions
- Articles published before January 1, 2010

The first exclusion criterion was added to identify barriers and facilitators that would be applicable to DMHIs. For example, a study that tests an app primarily focused on physical pain symptoms, with a mental health component, may find physical pain issues as a barrier to engaging with the app. It may not be clear from the study whether this is a common barrier related to DMHIs or interventions addressing physical pain.

The second and third exclusion criteria were added, as these types of interventions were designed to be a part of in-person sessions. It may not be clear from these studies whether users would be willing or able to engage with DMHIs apart from existing and traditional in-person sessions.

Finally, digital health interventions evolve rapidly [17,18], and the review was focused on the current state of DMHIs. Therefore, to avoid discussing on interventions or technologies that are now potentially out of date, the review was limited to contemporary studies published within the last 10 years (January 2010 to December 2019), a time frame that has been applied previously for systematic reviews on digital health technologies for mental illness [18].

### Search Strategy

A literature search was conducted in multiple databases, including SCOPUS, PubMed, PsycINFO, Web of Science, and the Cochrane Library. On the basis of the inclusion criteria, a search query was developed to include an article if its title or abstract contained at least one keyword related to mental health, at least one keyword related to digital interventions, and at least one keyword related to user experience (Textbox 3; PRE/5 means that keywords were separated by a maximum of 5 words, for example, online PRE/5 intervention means there were 5 or less words between *online* and *intervention*).

The search query was built on keywords used in previous reviews on the uptake of mental health technologies [11,19], and additional keywords were added for the specific focus of this review (ie, the third part of the query with keywords related to user experience). The search terms for each database are included in Multimedia Appendix 1. Searches were not limited to the study design.

**Search query.**

TITLE-ABS-KEY ( depress* OR anxiet* OR anxious OR mood OR “mental health” 

OR “psychological wellbeing” OR “mental wellbeing” OR “behavioral health” OR 

“mental illness” ) 

AND TITL E-ABS-KEY ( ( online PRE/5 intervention* ) OR ( online PRE/5 

treatment ) OR ( digital PRE/5 intervention* ) OR ( digital PRE/5 treatment )

OR ( mobile PRE/5 intervention* ) OR ( mobile PRE/5 treatment ) OR ( smartphone PRE/5 intervention* ) OR ( smartphone PRE/5 treatment ) OR ( web-based 

PRE/5 intervention* ) OR ( web-based PRE/5 treatment ) OR ( internet PRE/5 

intervention* ) OR ( internet PRE/5 treatment ) OR ( computer PRE/5 intervention* ) OR ( computer PRE/5 treatment ) OR ( cyber PRE/5 intervention* ) OR ( cyber PRE/5 treatment ) OR ( electronic PRE/5 intervention* ) OR ( electronic PRE/5 treatment ) OR ( mobile AND program* ) OR mhealth OR ehealth OR mtherap* OR etherap* OR telehealth OR telemedicine OR “mobile app*” )

AND TITLE-ABS-KEY ( usability OR “user experience” OR evaluation* OR

engagement OR interface OR satisfaction OR usage OR adoption OR acceptability OR qualitative OR user perspective* OR barrier* OR interview* OR focus group* )

### Study Selection

The search results were uploaded to Rayyan [20], a web-based software program for facilitating systematic reviews. Titles and abstracts were screened against the inclusion criteria, and excluded articles were labeled with reasons for exclusion.

The first author reviewed all titles and abstracts. Explicit inclusion criteria were determined between the first 3 authors a priori article selection to reduce coder bias. The coder (JB) was a PhD researcher with years of research expertise in user experience and thematic analysis.

A total of 6146 papers were extracted for the review. After the removal of 77 duplicates, 6069 article titles and abstracts were screened by the first author and discussed with the second and third authors. Uncertainties about inclusion were resolved by discussion among the first 3 authors, and reasons for exclusion or inclusion of these articles were discussed.

**Figure 1:**
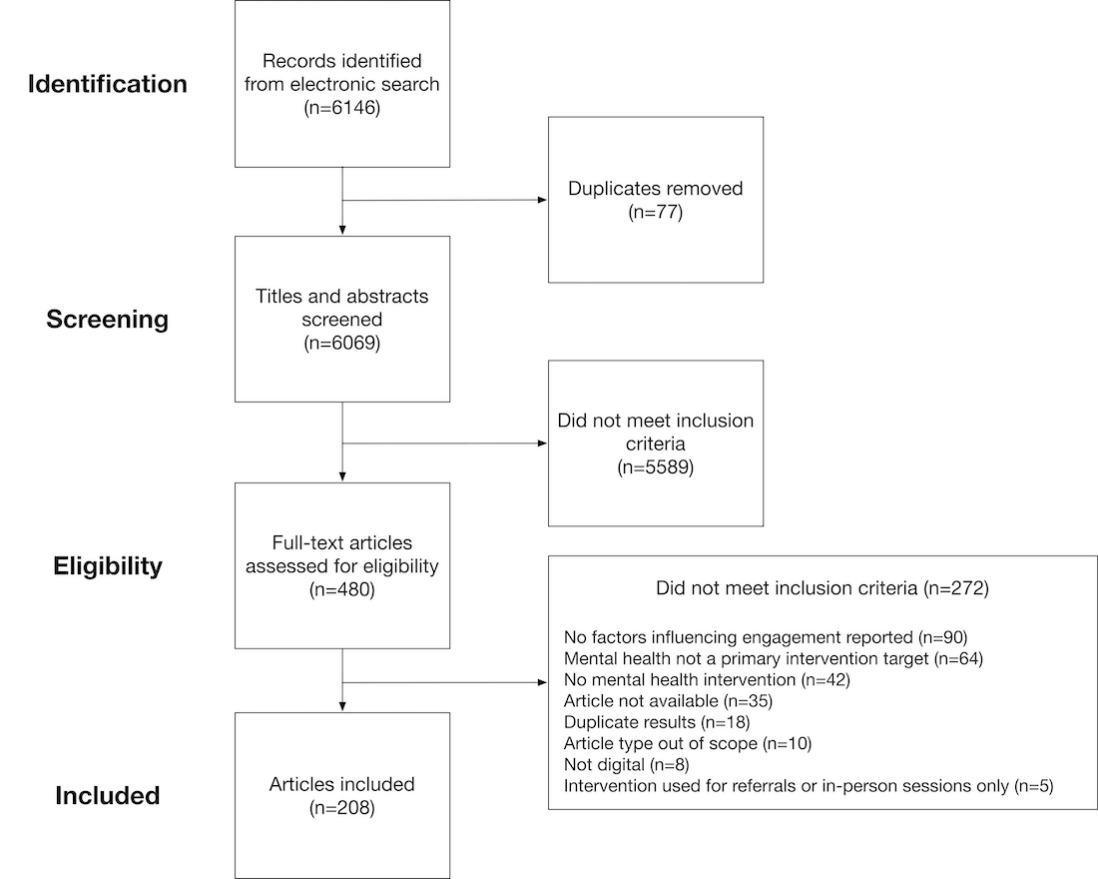
Flow diagram of article screening and inclusion.

Furthermore, 480 full-text articles were reviewed, of which 208 met the inclusion criteria. Figure 1 shows a flow diagram of the screening papers. The same inclusion and exclusion criteria were used for reviewing the titles and abstracts in the screening phase and reviewing the full-text articles in the eligibility phase.

Articles that were not available were either not available on the web or were behind a paid firewall. Article types that were out of scope did not report on an empirical study.

Although there is a risk of bias in studies, the review considered all studies that met the inclusion criteria and included a large variety of different study methodologies, including qualitative studies with no reported quantitative outcomes. The primary focus of this review was to establish themes across the literature rather than extract the outcomes of quantitative studies. Therefore, the risk of publication bias with significant results is small compared with a meta-analysis of outcomes [21].

### Data Extraction

A data extraction template (Multimedia Appendix 2) was developed for this review and piloted on 5 full-text papers. The main data elements extracted included reported factors, barriers, and facilitators to use and usage, such as retention and/or completion rate of the research study. The data were used to address the objective of the review to identify barriers and facilitators that influence user engagement.

Other extracted data were intended to document study and intervention characteristics, such as the type of technology and whether the intervention was publicly available, the target population, and the length of time that participants were able to engage with the intervention during the study.

### Quality Assessment

To account for the methodological variety of studies, the quality of reporting tool by Carroll et al [22] was used to assess quality. This tool has been used earlier in systematic reviews that include qualitative and quantitative studies [23]. Using this tool, articles were assessed on 4 criteria: (1) was the study design explained, (2) was the recruitment and selection of participants explained (eg, random sampling and convenience sampling), (3) were details of the data collection method provided (eg, topic guides for interviews, number of items in a survey, use of open or closed items), and (4) were details of the analysis method provided (ie, form of analysis rather than merely reporting data were analyzed). Following the tool’s guidelines, studies were considered to be *adequately reported* if a “yes” was assigned to 2 or more of these criteria.

### Analysis

An inductive thematic analysis [24] was used to identify common themes among these factors. This means that no preexisting coding scheme was used; rather, codes were created based on what emerged from the data.

We used a single coder approach, in which the first author iteratively identified codes from the data and refined themes throughout the analysis. Single coder approaches are methodologically sound when they include checks on validity and reliability [25]. For our analysis, validity and reliability were assessed by reviewing a selection of codes and their corresponding text with the second and third authors and by refining the codes. This process is common in qualitative research [26]. As we used emergent coding and there was no a priori codebook, a single coder approach also allowed for consistency of coding and interpretation of codes, and this approach has been used in systematic reviews [21].

The first author began the analysis by systematically reviewing each paper. For each paper, the following sections were analyzed: abstract, results or findings, and discussion. Individual codes were created each time a factor was described that affected engagement with DMHIs.

Factors were considered a barrier or facilitator if it was explicitly defined as a facilitator or barrier by the authors of the paper and/or the description in the paper pointed to it being a barrier or facilitator. For example, “participants reported they did not use mental health apps because they had privacy concerns on what would happen with their information.” In this instance, privacy concerns are identified as barriers.

A spreadsheet was used to keep track of the emerging codes. Each spreadsheet row corresponds to a single paper. The row contains the raw text of the paper that includes the identified factors and the initial codes. These codes were iteratively reviewed and compared with the raw text they were extracted from. Codes that referred to similar concepts, such as the ability to *personalize an intervention* and *customize an intervention*, were grouped together and given more descriptive names. As an understanding of the data was developed, earlier data were revisited to refine and combine codes, revalidating the previously coded material. Finally, the final codes were grouped into broader themes (eg, the roles of *age*, *gender*, and *employment* status were grouped into the theme demographic variables).

## Results

### Study Characteristics

As seen in Multimedia Appendix 2, the 208 articles included in this review [27-237] reported on 2 types of user studies: (1) 69 studies were needs assessments that aimed to understand user needs and attitudes toward DMHIs without or before engaging with a specific intervention as part of a study and (2) 135 studies were evaluation studies that assessed users’ experience with a specific intervention over the course of the study. In total, 4 articles included both needs assessment and evaluation. Overall, 35 articles explored general user attitudes about DMHIs without focusing on a specific technology, whereas 173 studies focused on a specific technology (Table 1). Although all studies involved interventions for mental health, some studies focused on a particular area: 45 studies focused on depression, 22 studies focused on stress, 9 studies focused on anxiety, 6 focused on eating disorders such as bulimia nervosa, 4 studies focused on mood, and 2 studies focused on loneliness.

**Table 1:** Type of technology studied in included articles.

Type of technology	Values, n (%)^a^
Web based	80 (38.5)
Smartphone based	57 (27.4)
Computer based, but not web based	9 (4.3)
Mobile phone (but not a smartphone)	5 (2.4)
Wearable technology	2 (1.0)
Tablet based	2 (1.0)
Combination of technologies	18 (8.7)

^a^Not all studies mentioned a particular treatment; hence, the percentages do not add up to 100%.

Measures related to user engagement included time spent using an intervention, number of log-ins, usability, acceptability, and feasibility. The usability and acceptability of the technology were assessed using qualitative methods and standard measures, such as the survey based on the Unified Theory of Acceptance and Use of Technology [27], the Mobile Application Rating Scale [28], and the System Usability and After-Scenario Questionnaire [29]. Feasibility was defined in these studies as either completion of a program offered through the intervention or retention rate, which is the number of people who completed the research study as a proportion of the people who started the study. In total, 42 studies employed qualitative interviews to understand people’s user engagement.

Factors that influenced user engagement were assessed through surveys (72/208, 34.6%), interviews (42/208, 20.2%), focus groups (34/208, 16.3%), randomized controlled trials (23/208, 11.1%), field studies (8/208, 3.8%), workshops (3/208, 1.4%), analysis of app usage data (7/208, 3.4%), and analysis of user reviews (2/208, 1.0%), using both qualitative and quantitative methods. For example, qualitative methods gathered subjective user perceptions of what formed barriers and facilitators for them to engage with interventions. A quantitative approach explored associations between variables, such as sociodemographic factors and intervention usage data, user satisfaction, and/or interest in using DMHIs.

The number of participants involved in these studies ranged from 6 to more than 2 million. In total, 6 studies conducted a secondary analysis of the usage data of an existing intervention or health database. For these 6 studies, the sample size was relatively large, ranging from 3158 to 2,171,325 users. Among the remaining 202 studies, the sample size ranged between 6 and 1558 users. For instance, 25% (52/208) of the studies had <18 participants, 49.5% (103/208) had <40 participants, and 75% (156/208) had <177 participants. The extent to which participants were exposed to an intervention ranged from a short demonstration before a focus group or survey to up to 1 year of usage.

### Quality Assessment

All studies were assessed as *adequately reported* (Multimedia Appendix 3 [143,172]). Each study reported on the research question, study design, and method of data collection. Overall, 11 studies did not report the recruitment and/or selection process of study participants [30-40]. In addition, 11 studies did not specify the analysis method used to analyze the data [33,41-50]. One study reported on the analysis method for the quantitative data that were collected but not qualitative data [51].

### Intervention Characteristics

Table 2 shows the types of technologies studied in the articles, and Table 3 shows the types of treatments and/or resources offered by the technology. Web- and smartphone-based interventions were the most common, reported in 38.5% (80/208) and 27.4% (57/208) of the papers, respectively. The most common type of treatment is internet-based CBT. Other treatments and features included acceptance and commitment therapy, psychotherapy, positive psychological interventions, meditation, peer support, resources, monitoring of symptoms, and journaling.

The target population included students, transitional age youth (aged 16-24 years), refugees, people who were homeless, veterans diagnosed with post-traumatic stress disorder, mothers with postpartum depression, patients being treated for a mental illness or another health concern, older adults, and caregivers and workers experiencing stress. Not all interventions specified the target population.

**Table 2:** Type of technology studied in included articles.

Type of technology	Values, n (%)^a^
Web-based	80 (38.5)
Smartphone-based	57 (27.4)
Computer-based, but not web-based	9 (4.3)
Mobile phone (but not a smartphone)	5 (2.4)
Wearable technology	2 (1.0)
Tablet-based	2 (1.0)
Combination of technologies	18 (8.7)

^a^Not all studies mentioned a particular treatment; hence, the percentages do not add up to 100%.

**Table 3:** Type of treatment and resources offered.

Type of treatment or resources	Values, n (%)^a^
Cognitive behavioral therapy	30 (14.4)
Informational or educational resources	23 (11.1)
Counseling	17 (8.2)
Self-tracking tools (eg, journaling, monitoring symptoms)	12 (5.8)
Mindfulness	9 (4.3)
Acceptance and commitment therapy	8 (2.9)
Peer support (eg, peer chat)	7 (3.4)
Text messaging (eg, reminders)	4 (1.9)
Positive psychology interventions	3 (1.4)
Prolonged exposure therapy	1 (0.5)
Passive data collection	1 (0.5)
Combination of treatments and/or resources	40 (19.2)

^a^Not all studies mentioned a particular treatment; hence, the percentages do not add up to 100%.

### Constructs Associated With User Engagement

Textbox 4 shows the high-level constructs derived from the thematic analysis influencing user engagement with DMHIs, where the numbers in parentheses show the number of articles in which the constructs were identified. We caution that the most frequently occurring constructs are not necessarily the most *important* but rather indicate that more studies have reported on this topic. Table 4 summarizes the main findings associated with each construct. After several iterations of grouping and coding, 16 larger groups remained: demographic variables, personal traits, mental health status, beliefs, mental health and technology experience and skills, integration into life, type of content, perceived fit, perceived usefulness, level of guidance, social connectedness, impact of intervention, technology factors, privacy and confidentiality, social influence, and implementation. These themes fit into 3 categories: user-related factors, program-related factors, and factors related to the technology and implementation environment. The next section provides more detailed explanations. The full list of factors belonging to each construct is included in Multimedia Appendix 4.

**The constructs influencing user engagement, grouped as constructs related to the user, the program offered by the intervention, and the technology and (implementation) environment. The numbers in parentheses indicate the number of articles in which the constructs occurred.**

User

- Demographic variables (31)
- Personal traits (5)
- Mental health status (59)
- Beliefs (55)
- Mental Health and Technology Experience and Skills (33)
- Integration into life (42)

Program

- Type of content (54)
- Perceived fit (61)
- Perceived usefulness (35)
- Level of guidance (40)
- Social connectedness (53)
- Impact of intervention (62)

Technology and environment

- Technology factors (100)
- Privacy and confidentiality (47)
- Social influence (16)
- Implementation (39)

**Table 4:** Summary of findings for each construct.

Construct		Summary of main findings
**User-related constructs**		
	Demographic variables (sociodemographic factors, such as age, gender, and education)	Overall, women were more likely to engage with DMHIs^a^ than men
	Personal traits (factors related to personality traits, such as neuroticism and extraversion)	The personality traits neuroticism, agreeableness, openness, and resistance to change were associated with higher engagement, whereas extraversion was associated with lower engagement
	Mental health status (factors related to the current mental health status of the user, such as the type and severity of symptoms)	Severity of mental health symptoms increased the interest in DMHIs, but symptoms related to depression, mood, and fatigue were a barrier to actual engagement
	Beliefs (beliefs held by the user with regard to technology, mental health, and mental health services)	People’s positive beliefs about mental health help-seeking and technology-facilitated engagement
	Mental health and technology experience and skills (previous experience the user has had with technology, mental health technology, and mental health services and skills related to their digital or mental health or digital health literacy)	Digital health literacy and positive experiences with mental health services and technology were facilitators to engagement
	Integration into life (the extent to which the user is able to find time and space to use the intervention and make the intervention part of their routine or life)	Engagement was facilitated if people were able to integrate DMHI use into their daily lives
**Program-related constructs**		
	Type of content (the type of content and features offered by the intervention)	Engagement was facilitated if content was credible and if activities offered by the DMHI were of an appropriate length (ie, not too short or too long)
	Perceived fit (factors related to how well the intervention is appropriate to the user’s culture and values and is adaptable to the user’s needs rather than a one-size-fits-all solution)	Engagement was facilitated if information offered by a DMHI was customizable and relevant to the user
	Perceived usefulness (factors related to expected benefits of using the digital intervention over existing resources)	Participants were more likely to engage with DMHIs if they understood the data and knew how to use it
	Level of guidance (the level of guidance offered by the intervention on how [eg, when, how often] to use it, for example, through notifications or a coach)	Guided interventions, either through a human therapist or automated reminders to use a DMHI, had higher engagement than unguided interventions
	Social connectedness (the extent to which the intervention connects or isolates the user with or from others)	Being able to connect with other people through a DMHI facilitated engagement
	Impact of intervention (the impact that intervention usage had on the user, such as an improvement or exacerbation of mental health symptoms [as measured by a validated survey scale])	DMHI engagement was facilitated if participants experienced a positive impact as a result of using a DMHI, such as the improvement of symptoms
**Technology- and environment-related constructs**		
	Technology-related factors (factors related to the technology through which the intervention is offered, such as the resources and costs required to use it, usability, and technical issues experienced by the user)	Technical issues were a common barrier to engagement
	Privacy and confidentiality (factors related to data security, storage, confidentiality, and privacy of the digital intervention)	Engagement was facilitated if participants had a sense that the digital platform was private and anonymous, and they could safely disclose information
	Social influence (factors from the users’ social environment, such as perceptions held by their peers, family, and health care provider, that influence their intention to use an intervention)	Participants were more likely to use DMHIs if people close to them, such as family and friends, thought they should use DMHIs
	Implementation (factors related to the implementation of the intervention that affects use, such as the availability of user training, the phase of the user’s mental health care–seeking process during which the intervention is introduced or accessed and characteristics of the health care organization supporting the DMHI)	DMHI engagement was facilitated if people were trained on how to use it

^a^DMHI: digital mental health intervention.

### User-Related Constructs

User-related factors refer to factors related to the user, such as personal beliefs, skills, and experiences.

#### Demographic Variables

Some demographic variables were found to be associated with DMHI engagement. Studies that found an effect of gender showed that women were more likely to adopt and engage with interventions [44,52-68]. Overall, 8 studies saw an effect of age: 2 studies found that people aged ≤50 years engaged more with interventions than older adults [66,67]. These 2 studies used a relatively large sample size (1,139 and 2,171,325), and participants were exposed to the intervention for up to 1 year. A total of 6 studies found that higher engagement was seen with adults aged ≥30 years [54,57,64,65,69-71]. These studies had a smaller sample size (samples ranged from 74 to 577 people), and participants engaged with the intervention for shorter periods (up to 12 weeks). Age was also found to influence interest and expectations: users’ interest in using digital therapy interventions increased with age [72], and Krause et al [58] found that older people have higher expectations of interventions.

Chudy-Onwugaje et al [73] found that age has an interacting effect with people’s depression symptoms. For people aged ≤40 years, adherence increased with depressive symptoms, but there was no association between depressive symptoms and adherence in people aged >40 years. Although the reasons for this interaction were unclear from the study, the authors of the article theorize that the effect of symptoms may interact with familiarity with technology, with younger people being more comfortable using technology.

Other demographic variables associated with user engagement were as follows: (1) employment status, with people who worked full time more likely to use the intervention than people who were retired [66] or unemployed [54,68]; (2) education, with participants with higher education reporting more acceptance of interventions than people with lower education (a high school diploma or lower) [74-76]; and (3) housing situation, with people who were experiencing homelessness responding less to messages sent by a phone intervention compared with individuals with stable housing [55].

#### Personal Traits

Certain personality traits were associated with willingness and interest in using DMHIs. People who scored high on neuroticism and agreeableness of the Big 5 personality traits were more interested in using smartphone apps to reduce stress [77]. In a different survey reported in the same article, neuroticism was strongly linked to self-reported stress. The cooperative nature of agreeable people made it easier to accept new technology.

In addition, extraversion was a predictor of lower likelihood to prefer web-based mental health services over in-person services [72]. People who scored high on extraversion preferred to meet and connect with a doctor in person. Other personality characteristics associated with user engagement were resistance to change and openness to experience [56]. Higher openness predicted higher engagement with mindfulness and relaxation interventions. Contrary to the hypothesis made by the authors of the article that higher resistance to change would lead to resistance to adopting a new health behavior, higher resistance instead predicted higher adherence. Once people started using the intervention, a higher resistance to change facilitated commitment to continue using the intervention.

#### Mental Health Status

A total of 59 studies reported that people’s mental health status plays a role in participants’ interest in and use of a digital intervention. First, certain mental health symptoms appeared to inhibit people’s motivation and/or ability to interact with an intervention. Depressive symptoms [78] and low mood [79], as measured by validated scales, have been reported as barriers for people to access and use web-based resources. Study participants reported that feeling tired also negatively affected their motivation and ability to use an intervention [44,80]. Second, the severity of these symptoms was related to engagement with digital interventions. In needs assessment studies, participants were more willing to use DMHIs if their symptoms were more severe [38,53,62,71,81,82]. However, evaluation studies have shown that more severe symptoms hamper actual engagement with digital interventions [51,56,83-101]. Depending on the type and severity of a person’s mental health symptoms, studies that involved health care providers supporting digital intervention use reported that there was sometimes a need for face-to-face contact, as issues could be difficult to address remotely via a digital platform [102-104].

#### Beliefs

Beliefs refer to preexisting beliefs the user has about mental health help-seeking [88], their need for help [51,105-107], the acknowledgment of having mental health needs [88], and using technology for mental health treatment [38,93,108,109]. For example, preexisting beliefs of needing help for mental health needs and having a positive perception about mental health help-seeking facilitated participants’ engagement with an intervention. However, even if people acknowledged a perceived need for help and were willing to seek help, engagement with a particular intervention was then affected by a person’s preconceived belief about whether a digital intervention would be effective [79,104,110-112]. In 2 studies, participants did not want to use a digital intervention because technology was seen as a stimulant and distracting [113,114].

#### Mental Health and Technology Experience and Skills

A positive prior experience with technology [115,116], mental health services [54,74,104,117-119], and mental health technology [72,120,121] facilitated people’s intention to use interventions, as well as actual engagement. A negative prior experience formed a barrier to engaging with a DMHI [74,117,122], whereas a positive experience increased participants’ engagement [54,72,104,115,116,118-121].

Mental health literacy refers to knowledge about mental health symptoms and appropriate treatment options [238]. Digital literacy refers to the skills required to use technology [239]. Digital health literacy refers to the ability to use technology to find and use health resources [240]. Participants’ mental health literacy [123], digital literacy [88,103,104,122,124-127], and digital health literacy [103,127] influenced the extent to which they were able to adapt and engage with DMHIs: for each type of literacy; higher literacy was associated with higher engagement.

#### Integration Into Life

Users reported that their engagement was affected by the extent to which they were able to integrate an intervention into their daily lives. Barriers that limited use included that participants felt they lacked time [44,128-131] or constantly forgot to use an intervention [93,95,129], participants felt the intervention took too much time to use [132-134], and participants experienced difficulties establishing a routine of use that worked for them [130,135].

Access to a private space to access mental health resources also affected the extent to which participants could integrate an intervention into their lives. In 3 studies, participants mentioned that as opposed to going to a health care provider office, it was challenging to find a private space at home or work to use an intervention, which formed a barrier to engaging with it [136-138].

Studies have also found difficulties among users in integrating the information and tips offered by the intervention into their lives. For example, Jonathan et al [139] evaluated a smartphone app for people with serious mental illness. Participants who spent most of their day indoors without leaving their house had a hard time trying to use the tips in actual real-life scenarios.

#### Summary of User-Related Constructs

In summary, user engagement with DMHIs is partly influenced by factors related to the users themselves. Demographic variables such as age, gender, employment, education, and housing situation can affect user engagement. The personality traits neuroticism, agreeableness, openness, and resistance to change facilitated engagement, whereas extraversion was a barrier.

If mental health symptoms were more severe, participants were more interested in using DMHIs, but symptoms related to depression, low mood, and tiredness prevented engagement. People’s beliefs about and past experiences with mental health services and technology were facilitators if these beliefs and experiences were positive, and they formed a barrier if these beliefs and experiences were negative. Participants’ literacy in understanding mental health and using technology facilitated their ability to use DMHIs, and any further engagement depended on the extent to which people were able to integrate it into their daily lives.

### Program-Related Constructs

The second group of constructs is related to the type of therapy or content offered through the DMHI.

#### Type of Content

Higher satisfaction with the type of content and features offered increased user engagement. Uncertainty about the credibility of the information, which related to the evidence base of the intervention and the source of information, was a barrier [74,88,127,140-143]. Other factors related to the modality through which content was delivered, with some participants preferring to have audio or video options in addition to text-only information [144] and whether the content was considered by users to have a supportive, nonjudgmental tone [51,145,146].

Some interventions offered programs of a fixed length or time commitment, such as a CBT program consisting of 8 weekly sessions. The length of the program as well as the length of individual sessions played a role in participants’ satisfaction and their motivation to continue with the program [88,138,147-151]. In 2 studies evaluating a self-guided CBT program for 8 weeks, the length and pace of modules negatively impacted user motivation [88], and participants reported preference for more concise modules [148], although the articles did not attempt to identify an ideal module length. In other studies evaluating a CBT program that included in-person sessions with a therapist, some participants reported preference for both longer individual therapy sessions (greater than the standard 50-70 minutes) [151] and duration of treatment to maximize benefit [150].

#### Perceived Fit

Perceived fit refers to the extent to which users felt the intervention was appropriate and relevant to their culture and values and/or targeted to people similar to them, rather than a one-size-fits-all solution. This fit was, for example, facilitated by relevance of information to their current situation [14,46,150,152-157] and the ability to customize or personalize the intervention [30,46,83,84,122,134,135,138,158-165]. A facilitating factor was whether users were able to identify with the people presented in the intervention [166], which could be coaches or instructors, or examples of people with similar experiences. Factors that make the information relevant and in a language suitable to the user included culturally appropriate content [133,167,168], reading level suitable to the user [168], and content presented with limited jargon or technical language [169].

#### Perceived Usefulness

Perceived usefulness refers to the user’s experience with an intervention and their perceptions of whether the intervention would be useful to them. This perception was facilitated by whether users were able to understand the data presented to them [104,117,170], whether it was clear what action they should take [129,133,154,155,166], and whether the intervention provided a clear advantage over past or current care received [103,117,121,155,171]. Identified facilitators were easier access to services that users would otherwise not have access to [103,173] and the eliminated need to travel a long distance to a health center [121].

#### Level of Guidance

The level of guidance refers to the extent to which users were guided to use an intervention, for example, through reminders or a web-based supporter, holding them accountable to regularly engage with the content. A facilitating factor in using DMHIs was whether the use of the intervention increased locus of control, meaning that users felt more ownership over their own health [14,84,95,124,174,175]. However, for interventions that were completely self-guided, participants experienced difficulty engaging with them and at times neglected to use the intervention [44,95]. Participants expressed a need to receive more structured use, for example, through app reminders or a human coach checking on them on a regular basis [49,50,113,122,133,137,139,148,150,163,175-182]. In 6 studies, users stated that they would prefer if an intervention served as a complement to existing, in-person therapy rather than a replacement for in-person therapy [30,122,134,139,166,183].

#### Social Connectedness

The effect that an intervention had on participants’ sense of social connectedness was found to facilitate user engagement. For example, being able to connect to peers or have regular contact with a personal therapist through DMHIs facilitated engagement in 18 studies [14,32,33,104,114,122,125, 133,156,184-194]. In 6 studies, a noted barrier among both users and service providers was a concern about social avoidance, that is, a concern that people might use self-guided interventions in lieu of coming into a clinic in person and engaging in therapy or group sessions [78,104,122,133,138,195]. For therapy interventions, where study participants were introduced to therapists who they did not know before using the intervention, the extent to which participants could connect emotionally (therapeutic alliance) with the therapist-influenced engagement. Participants’ ratings of the quality of the emotional connection were positively related to the number of log-ins, frequency of self-monitoring mood, and completion of therapy [196].

#### Impact of Intervention

Participants reported that the perceived changes they experienced in their mental health as a result of using an intervention affected their further engagement. Perceived symptom improvement facilitated further engagement [89,95,103,145,146,189,197-199], whereas exacerbation of symptoms negatively impacted engagement [49,93,104,200]. Other negative impacts of the intervention were also observed as barriers to ongoing user engagement. For example, in 2 studies, some information that was shared within the digital intervention was found to trigger difficult memories or emotions [150], participants were uncomfortable with exercises or information [80], or participants were exposed to negative comments by other users [150].

Another facilitating factor was whether the intervention normalized people’s experiences [79,139,157,190,201], for instance, by providing examples of other people with similar experiences. Additional positive effects that could facilitate engagement were an increased insight into users’ health [79,84,114,124,134,138-140,158,176,202-205], a feeling of empowerment over being in control of their health [14,32,84,93,95,104,117,122,124,170,174,175,196,206,207], improved skills [89,189,198,208-211], such as managing negative emotions, and an improvement of participants’ existing relationships with others [182,205].

#### Summary of Program-Related Constructs

In summary, the content offered by a DMHI had to be credible and ideally offered in more than one modality. Participants engaged with DMHIs if they felt the intervention was a good fit, which could be facilitated if content was relevant, and the DMHI was customizable, culturally appropriate, and used a language that was understandable to the participant. Engagement was facilitated by participants’ perception of whether a DMHI was useful, which included whether they were able to understand the data and how to use it, and whether a DMHI provided a clear advantage over resources they already had access to.

Guided DMHIs had higher engagement than unguided interventions, and participants liked being able to connect with other people, although some studies identified concerns that DMHIs could be used to avoid in-person contact. The negative and positive impacts of DMHI use could form barriers and facilitators, respectively, to further engagement.

### Technology- and Environment-Related Constructs

The third group of constructs refers to factors related to the technology itself or the implementation of the technology.

#### Technology-Related Factors

Technology-related factors refer to factors related to the technology through which the intervention was offered. The primary barrier to engagement noted in 25 studies was users’ experience of technical issues [44,50,80,92,100,103,118, 129,138,155,172,179,185,195,205,208,212-220], such as a mobile app crashing and shutting down unexpectedly; in 3 studies, participants did not have the resources required to use an intervention [171,221,222]. In 7 studies, participants expressed concerns over the eventual costs associated with using an intervention [85,93,104,123,165,223,224]. Costs could be related to the need for a smartphone, having internet access, or making purchases through the app. Usability issues formed a barrier to engaging with an intervention [46,50, 78,84,148-150,157,159,170,224-228]. Examples of usability issues were difficulty finding information in an intervention [78], a time-consuming process to log in to an intervention [159], and difficulty navigating within an intervention [150,157].

In addition to technical issues that formed barriers to engagement, there were also factors related to technology that facilitated the use of mental health resources and support. Facilitating factors made possible by the technology used were the flexibility of being able to access resources at any location [47,127] at any time [41,93,97,124,129,134,167,229-233] and having a temporal record of health data, such as symptoms, that users were able to track and access over time [176,180].

#### Privacy and Confidentiality

Privacy and confidentiality relate to how data were stored and shared and whether users felt safe and comfortable to disclose confidential information through an intervention. In 2 studies, participants were uncomfortable about their physical location being recorded [180,234], and in a study by Nicholas et al [234], participants were more comfortable with health information being recorded such as sleep and mood than personal data being recorded such as social activity and communication logs.

Accessing mental health resources via a digital platform raised concerns regarding privacy. Facilitators of user engagement and feeling safe to disclose information included assurance that the digital platform was private and participants’ information could not be easily accessed by third parties [129,158,205,235].

Participants in 5 studies expressed that concerns about confidentiality formed a barrier to engagement [51,104,127,236,237]. A facilitator to create a safe environment was moderation of the intervention [140], which means that a person was monitoring and moderating the content shared by and between users within an intervention.

Anonymity was found to be both a facilitator and a barrier to engagement. Overall, 7 studies listed anonymity, meaning that users could share and receive information anonymously, as a facilitating factor to engage and encourage disclosure of information [41,88,129,137,141,148,232]. However, anonymity could also make it more difficult for participants to trust a coach who they did not know [85,127]. In these studies, participants interacted with the coach through text, and there was the option to disclose names, but neither side could see each other. Other study participants were concerned about whether an intervention was truly anonymous if it was used in a small setting, with a limited number of known users [137]. Anonymity was also more important for people who were older and who had previous experience with medical treatment [127].

#### Social Influence

Users’ engagement was facilitated by whether the intervention was endorsed by other users [43] or peers [211,241], their friends and family [97], or their current health care provider [152,161,242]. However, if participants felt forced by others to use an intervention, it deterred them from using it [103]. If an intervention was used as part of ongoing in-person therapy, the way that therapists used or were willing to use an intervention influenced participants’ engagement with an intervention [84,103,127,150,169,212,243]. The adoption of an intervention as part of therapy depended on the therapist’s digital literacy skills [244,245], their past experience with mental health technology [120], and the ability to easily integrate its use into their practice as a provider [132,195,205,211,224,226,246].

#### Implementation

Although most studies in this review (93%, 194/208) primarily focused on factors related to the user and the intervention itself, 14 studies also described factors related to the implementation of the intervention. Examples included whether users received training on how to use the intervention [115,247] and if it was introduced early on or at a later stage in ongoing therapy. Participants in a study by Graham et al [68] used an intervention to support their mental health while in treatment for substance use. These participants found the intervention more useful at a later stage, as they felt the user was likely more familiar with their health and better able to make sense of the information provided by the intervention. Two other studies found that participants engaged more with an intervention if they were just starting treatment [104,178]. Two studies found that the way in which the intervention was labeled and introduced to users also mattered. For example, the term *mental health* was disliked by participants [248], and participants reported that they would be more likely to use an app if it was meant for *well-being* and *mental fitness* rather than mental health [37]. Other implementation factors were administrative barriers [42,118,129,211] and barriers related to the organization in which the intervention was or would be implemented [118,122,135]. Examples of administrative barriers were inadequate staffing and poor communication among staff members. An example of organizational barriers was a lack of support for DMHIs among managers.

#### Summary of Technology- and Environment-Related Constructs

In summary, although DMHIs introduced technical and usability issues that could form a barrier for participants to engage, the digital format also provided flexibility to access resources anywhere at any time and to have a record of health data. It was important that information was private and that participants could safely disclose information anonymously, although complete anonymity also made it more difficult to trust other people on the platform. Negative and positive opinions held by other people about DMHIs could form a barrier and facilitator, respectively, to engagement, and if DMHIs were to be used as part of ongoing therapy, the therapists’ past experience with DMHIs and the ability to integrate it into their practice played a role in user engagement. Finally, successful implementation facilitated user engagement. Providing training on how to use DMHIs and labeling an intervention for well-being or mental fitness (as opposed to mental health) can help users engage with DMHIs more. Participants may be more engaged with DMHIs if they are just starting treatment, but the identified benefit of introducing DMHIs at a later stage is that users may be more knowledgeable about their health and better able to make sense of their health information.

## Discussion

### Principal Findings

This study aims to synthesize the literature on DMHIs and summarize the identified factors affecting user engagement with DMHIs. This review identifies 3 key areas that all contribute to DMHI engagement: (1) user characteristics, such as severe mental health symptoms, can form a barrier to engagement; (2) users’ experience of the program or content, with participants more likely to engage if they perceive the program to be useful and a good fit to them; and (3) the technology and implementation environment, such as technical issues being a common barrier to engaging with DMHIs. Providing content that is relevant and customizable according to personal preferences and offering technical assistance and/or training are important to achieve engagement. However, although these considerations may increase interest and uptake of DMHIs, it is important to understand whether characteristics specific to the user, such as their symptoms, will affect motivation to engage with these interventions. We first discuss the 3 key areas in more detail in the following three subsections; compare our constructs with other models on user engagement; and then discuss implications for researchers, developers, and health service providers.

### User Constructs

Individual differences among users can affect engagement, including demographic variables such as age and gender, personality traits, mental health status, beliefs about mental health and DMHIs, experience with technology and mental health, and people’s ability to integrate DMHI use into their lives. Although the severity of symptoms may increase interest in engaging with health interventions [249], symptoms related to depression, mood, and tiredness were found to hamper actual engagement. This contrast may point to the unique implications that mental health symptoms can have on engagement with DMHIs.

The contrasting role of symptom severity between studies highlights the importance of understanding how people who would be more interested in DMHIs and may benefit more from its use are not limited by their symptoms to actually engage with these interventions. The contrast also illustrates the importance of including users at various stages of the design process, as people may be interested in the concept of a DMHI but may not be able to actually engage with it because of the nature of their symptoms.

Although studies looking at DMHI usage over 1 year found that younger people were more engaged with DMHIs, shorter research studies (ie, up to 12 weeks) found that older people were more engaged. Potentially, older adults perform better on study adherence, and younger people continue to engage more with an intervention long term, although the different interventions and settings make it difficult to make a direct comparison between these studies.

### Program Constructs

Engagement with DMHIs was facilitated if participants liked the type of content; they perceived a DMHI to be a good fit for them and perceived it to be useful; there was a level of guidance on how to use it, it facilitated social connectedness, and it had a positive impact, such as improvement of symptoms.

#### Level of Guidance

Guided interventions typically have higher engagement than unguided interventions. However, human guidance can be resource intensive, and it may not always be possible or feasible to provide the desired level of guidance. Although human support enhances engagement more than automated means such as email reminders [250], several studies included in our review found that such automated reminders not only facilitated engagement but were also experienced positively by users. Automated reminders to use an intervention may therefore be a low-cost alternative to human support. The benefits of automated reminders may depend on the type of support and the type of barriers they are designed to address. Short text-based reminders may be suitable for in-the-moment interventions [251] and may be useful to address barriers to forgetting to use an intervention. On the other hand, human support may be more suitable for addressing the lack of motivation and facilitating social connectedness.

Furthermore, appropriate time commitments differ for self-guided exercises versus guided sessions. Participants across studies preferred shorter self-guided modules but longer guided therapy sessions. Finally, personalization may also meet different preferences. People who find videos or text-based material time consuming may be more engaged with shorter actionable exercises, whereas people with a preference for synchronous communication may engage more when they get dedicated time on one-on-one sessions. It would be worthwhile to further explore how engagement can be encouraged in self-guided interventions.

#### Social Connectedness

An important facilitator was whether a DMHI facilitated social connectedness and enabled the user to interact with other people. Previous work has shown that social support through social networks not only increases engagement but may also have a positive effect on depression symptoms [221,222]. However, in some studies in this review, mental health service users and providers were concerned that technology would facilitate social avoidance if people were to use a digital intervention in lieu of engaging in face-to-face individual or group therapy. It appears that it is important that an intervention allows users to connect with other people with whom they may have otherwise not connected, rather than replacing any existing face-to-face contact. For example, people can access a mental health app if they are not able to speak to someone in person about their concerns [175].

### Technology and Environment Constructs

Offering mental health resources through technology offers both barriers and facilitators. Technical issues and concerns about privacy were common barriers, but technology also offered flexibility and could facilitate anonymity. Furthermore, the environmental context in which DMHIs are to be used are important to consider. Participants were more likely to use DMHIs if people close to them thought they should use it and if they received training on how to use it.

#### Anonymity

Anonymity was a prominent topic among studies but engaging with an intervention anonymously was seen as both a barrier to and facilitator of engagement, sometimes within the same study. This difference can be explained by factors related to the user, the type of implementation setting, and the type of intervention features that were anonymous, as outlined in the following paragraph.

First, a facilitating aspect of an anonymous intervention was that study participants found it less stigmatizing than seeing a live or in-person therapist. Anonymity may be an important facilitator for people who have experienced stigma and embarrassment, which is known to be a barrier to help-seeking for mental health concerns [2,3]. Similarly, prior work on mental health discourse on the web found that anonymity does not hinder the social support that people receive on their posts, which can facilitate open conversations, and that social media may be particularly useful for stigmatic illnesses such as mental health [252]. Second, the study setting matters. Interventions that are used in a relatively small setting may give a false sense of anonymity if it is possible for users to find out who else is using the intervention, for example, through content shared within the intervention or by seeing someone use it [108], which is important to consider for intimate settings, such as schools, workplaces, or small communities. Third, on community forums, where users could share their experiences and comment on other users’ posts, overall anonymity was seen as a facilitator to safely disclose information. In one-on-one sessions, however, where the user interacted with a coach or therapist but neither side could see each other, anonymity made it more difficult to establish a relationship and trust compared with a face-to-face session.

These differing perceptions shed light on an important trade-off. Should an intervention strive to be anonymous to address stigma and potential embarrassment or focus on allowing people to establish a trusted relationship with someone? This decision may depend on the objective of the intervention and whether anonymity is possible in the context in which it is to be used. Alternatively, a hybrid form or multiple options can be considered and offered. For example, forums with a larger number of users can be anonymous, whereas a private one-on-one session with a therapist can include telehealth options to allow for therapeutic alliance building between the user and therapist. The Supportive Accountability Model [250] also proposes that engagement is enhanced if human coaches are seen as trustworthy and that users may disclose more in computer-mediated than face-to-face communication. Although Mohr et al [250] argue that providing additional information about individuals, such as photographs, may reduce these types of benefits of this type of mediated communication, it may be important to establish initial trust with a coach or therapist. Additional research is needed to understand how best to support trust in DMHIs.

#### Privacy

A previous review of user engagement with mental health apps theorized that one reason for low engagement is that these apps do not consider user privacy [15]. In our review, privacy was discussed in terms of data storage and sharing but also with respect to the physical environment in which these interventions were to be used. Delivering mental health support through a digital platform was found to increase a sense of privacy in some studies, but in other studies, it was associated with a decreased sense of privacy. The study participants stated that they could access care more privately, without anyone knowing about it. In line with previous work [2], this again indicates that privacy can be important for people who experience stigma for or reluctance to help-seeking. Although participants’ living situation was not explicitly discussed in these studies, when compared with other studies, it is likely that participants were able to engage with these interventions in physically private settings. In other studies where people did not have access to a private space, a lack of privacy was a barrier to engagement. For example, study participants evaluating an app that delivered remote web-based therapy felt that they could disclose more in a closed therapist office than through a web-based intervention at home where other people in their household could see or disrupt them [136]. Study participants who used a mental health intervention in the workplace [137] said privacy was not possible, as colleagues could see what someone was doing at their desk and when they were interacting with the intervention.

These differing experiences highlight that technology can overcome existing privacy barriers of seeking mental health care but can also introduce other privacy issues, and users’ situational context (ie, where they are physically accessing the digital intervention) should be taken into account.

### Comparison With Other Models of Technology and Digital Health Intervention Engagement

Some of the themes identified in this review overlap with previous models conceptualizing engagement with digital health interventions, as well as general technology acceptance and health behavior, such as the Efficiency Model of Support [251], Technology Acceptance Model [253], and Health Belief Model [249].

For instance, the Efficiency Model of Support [251] states that human support increases engagement in the context of the use of digital health interventions when it addresses 1 of 5 failure points: *usability, implementation, fit, engagement*, and *knowledge*. These broadly map to our constructs of technology factors, integration into life, perceived fit, beliefs, and experience, and skills. The *implementation* failure point in the efficiency model pertained to whether the user can apply knowledge gained from an intervention in their lives. Our review extends this concept, in that we found that an important issue is whether users can integrate the actual use of the intervention into their everyday routines.

Our findings are in line with the Technology Acceptance Model, which explains that users’ decisions to accept and use a technology are influenced by perceived usefulness, ease of use, and social influence of others. The Health Belief Model explains that adoption of health interventions is, among things, influenced by a person’s belief in the severity of their illness or health symptoms and the perceived benefits of seeking treatment for these symptoms, which map onto our constructs of beliefs and impact of the intervention. Themes revealed in this review, which have not been highlighted in these previous models, are the level of guidance, integration into life, and social connectedness. This gap may be explained by the way in which mental health interventions were intended to be used. To be effective, most DMHIs were intended to be used regularly by users on their own. This characteristic introduces the challenge for people to integrate it into their routine and have the discipline to use it regularly; therefore, the level of guidance provided within the intervention may have a particularly salient effect on engagement. Social connectedness may be especially important for mental health interventions, as it can improve mood [254] and help combat depression [255].

### Implications

In this review, we have synthesized the literature on DMHIs to identify common factors influencing user engagement. This synthesis can be described as follows.

- Researchers can use these factors to develop constructs that are important to measure when evaluating DMHIs. More concretely, it is important to capture user characteristics, users’ experience of the program and content, and details regarding the implementation setting. These constructs may help explain why someone would use one DMHI over another and may help evaluate how engaging a DMHI will be.
- Developers can use these factors to facilitate engagement with DMHIs. Specifically, when developing a DMHI, it is important to understand the specific characteristics of the target audience, for example, if the severity of the audience’s symptoms can form a barrier to engagement; to tailor the program to the audience, such as offering the option to customize content; and to address issues related to the technology and environment, for example, by mitigating technical issues and providing technical assistance.
- Mental health service providers, such as clinicians, can use this overview as guidance to select interventions that are appropriate for their clients or help guide their clients in selecting suitable interventions. For example, it is important to consider whether an intervention can be easily integrated into clients' lives and routines. In addition,Multimedia Appendix 2, which shows the full data set, can be used to filter the study setting, target population, and symptoms to see which barriers and facilitators have been observed for similar settings and populations.

The themes highlighted in this review identify factors that can facilitate engagement and barriers that should be considered to facilitate the successful implementation of a digitally mediated mental health intervention.

### Limitations

We did not limit this review to particular study designs. As such, this review takes a much broader look at what factors influence engagement with digital mental health technologies rather than focusing on a single research method or technology. However, because of the heterogeneity of the included studies, we were unable to conduct a meta-analysis. In addition, there was inconsistency across studies in measures used to assess user engagement, such as the number of log-ins to an intervention, the length of continuing to engage with it, the total time spent using an intervention, or a self-reported measure of engagement by participants. This inconsistency has been found to be an issue in previous reviews on the user engagement of DMHIs [11,256]. This review was limited to peer-reviewed empirical articles. Although the review included articles that evaluated people’s experience with both research interventions and commercially available DMHIs, it is possible that some interventions may have been missed.

Finally, this review was conducted before the global COVID-19 pandemic. There may be unique factors that are pandemic related that make DMHI engagement more or less likely. For example, stay-at-home orders may exacerbate feelings of social isolation and make people more likely to engage with apps that increase social connectedness. On the other hand, it may also introduce additional barriers to finding a private space to use DMHIs if sheltering in place with others. The results presented in this review should be interpreted and used to understand DMHI engagement before and after the pandemic. A future review could be conducted solely during the pandemic period, and it could be compared with this review to understand DMHI use outside versus during a pandemic.

### Conclusions

Previous studies have shown the potential of DMHIs to improve mental health. However, for these interventions to be clinically effective, they require engagement by users in real-world settings. Across the studies reviewed, we identified 16 common factors that affect user engagement. Further research on DMHIs can use these factors as guidelines when evaluating interventions with users, and future interventions can be developed with these factors in mind. By understanding the factors that affect engagement, targeted strategies can be developed to overcome addressable barriers and work toward the successful implementation of these interventions.

## Appendix

Multimedia Appendix 1 Overview of search queries or terms used for each database.

Multimedia Appendix 2 Data extraction template with metadata of the reviewed articles.

Multimedia Appendix 3 Quality assessment of included studies.

Multimedia Appendix 4 Overview of barriers and facilitators for each theme.

## Abbreviations

CBT: cognitive behavioral therapy
DMHI: digital mental health intervention
