# Supplementary material for: Barriers to and Facilitators of User Engagement With Digital Mental Health Interventions: Systematic Review
Source: J Med Internet Res. 2021 Mar 24;23(3):e24387. doi: 10.2196/24387 (PMC8074985; doi:10.2196/24387)
Supplement: Multimedia Appendix 1 [file jmir_v23i3e24387_app1.doc]

# **Search Strategy Overview**

Period: 19 November-4 December 2019

## **SCOPUS**

TITLE-ABS-KEY ( depress*  OR  anxiet*  OR  anxious  OR  mood  OR  "mental health"  OR  "psychological wellbeing"  OR  "mental wellbeing"  OR  "behavioral health"  OR  "mental illness" )

AND  TITLE-ABS-KEY ( ( online  PRE/5  intervention* )  OR  ( online  PRE/5  treatment )  OR  ( digital  PRE/5  intervention* )  OR  ( digital  PRE/5  treatment )  OR  ( mobile  PRE/5  intervention* )  OR  ( mobile  PRE/5  treatment )  OR  ( smartphone  PRE/5  intervention* )  OR  ( smartphone  PRE/5  treatment )  OR  ( web-based  PRE/5  intervention* )  OR  ( web-based  PRE/5  treatment )  OR  ( internet  PRE/5  intervention* )  OR  ( internet  PRE/5  treatment )  OR  ( computer  PRE/5  intervention* )  OR  ( computer  PRE/5  treatment )  OR  ( cyber  PRE/5  intervention* )  OR  ( cyber  PRE/5  treatment )  OR  ( electronic  PRE/5  intervention* )  OR  ( electronic  PRE/5  treatment )  OR  ( mobile  AND program* )  OR  mhealth  OR  ehealth  OR  mtherap*  OR  etherap*  OR  telehealth  OR  telemedicine  OR  "mobile app*” )

AND  TITLE-ABS-KEY ( usability  OR  "user experience"  OR  evaluation*  OR  engagement  OR  interface  OR  satisfaction  OR  usage OR adoption OR acceptability OR qualitative OR user perspective* OR barrier* OR interview* OR focus group*)

## **PsycINFO**

(depress* OR anxiet* OR anxious OR mood OR “mental health” OR “psychological wellbeing” OR “mental wellbeing” OR “behavioral health” OR “mental illness”)

AND ((online PRE/5 intervention*) OR (online PRE/5 treatment) OR (digital PRE/5 intervention*) OR (digital PRE/5 treatment) OR (mobile PRE/5 intervention*) OR (mobile PRE/5 treatment) OR (smartphone PRE/5 intervention*) OR (smartphone PRE/5 treatment) OR (web-based PRE/5 intervention*) OR (web-based PRE/5 treatment) OR (internet PRE/5 intervention*) OR (internet PRE/5 treatment) OR (computer PRE/5 intervention*) OR (computer PRE/5 treatment) OR (cyber PRE/5 intervention*) OR (cyber PRE/5 treatment) OR (electronic PRE/5 intervention*) OR (electronic PRE/5 treatment) OR (mobile program*) OR mhealth OR ehealth OR mtherap* OR etherap* OR telehealth OR telemedicine OR “mobile app*”)

AND (usability OR "user experience" OR evaluation* OR engagement OR interface OR satisfaction OR usage OR adoption OR acceptability OR qualitative OR user perspective* OR barrier* OR interview* OR focus group*)

## **PubMed**

(depress* OR anxiet* OR anxious OR mood OR “mental health” OR “psychological wellbeing” OR “mental wellbeing” OR “behavioral health” OR “mental illness”)

AND ((online PRE/5 intervention*) OR (online PRE/5 treatment) OR (digital PRE/5 intervention*) OR (digital PRE/5 treatment) OR (mobile PRE/5 intervention*) OR (mobile PRE/5 treatment) OR (smartphone PRE/5 intervention*) OR (smartphone PRE/5 treatment) OR (web-based PRE/5 intervention*) OR (web-based PRE/5 treatment) OR (internet PRE/5 intervention*) OR (internet PRE/5 treatment) OR (computer PRE/5 intervention*) OR (computer PRE/5 treatment) OR (cyber PRE/5 intervention*) OR (cyber PRE/5 treatment) OR (electronic PRE/5 intervention*) OR (electronic PRE/5 treatment) OR (mobile program*) OR mhealth OR ehealth OR mtherap* OR etherap* OR telehealth OR telemedicine OR “mobile app*”)

AND (usability OR "user experience" OR evaluation* OR engagement OR interface OR satisfaction OR usage OR adoption OR acceptability OR qualitative OR user perspective* OR barrier* OR interview* OR focus group*)

## **Web of Science**

Topic ( depress* OR anxiet* OR anxious OR mood OR “mental health” OR “psychological wellbeing” OR “mental wellbeing” OR “behavioral health” OR “mental illness”)

AND Topic (online PRE/5 intervention*) OR (online PRE/5 treatment) OR (digital PRE/5 intervention*) OR (digital PRE/5 treatment) OR (mobile PRE/5 intervention*) OR (mobile PRE/5 treatment) OR (smartphone PRE/5 intervention*) OR (smartphone PRE/5 treatment) OR (web-based PRE/5 intervention*) OR (web-based PRE/5 treatment) OR (internet PRE/5 intervention*) OR (internet PRE/5 treatment) OR (computer PRE/5 intervention*) OR (computer PRE/5 treatment) OR (cyber PRE/5 intervention*) OR (cyber PRE/5 treatment) OR (electronic PRE/5 intervention*) OR (electronic PRE/5 treatment) OR (mobile program*) OR mhealth OR ehealth OR mtherap* OR etherap* OR telehealth OR telemedicine OR “mobile app*”)

AND Topic (usability OR “user experience” OR evaluation* OR engagement OR interface OR satisfaction OR usage OR adoption OR acceptability OR qualitative OR user perspective* OR barrier* OR interview* OR focus group*)

## **Cochrane Library**

Title Abstract Keyword ( depress* OR anxiet* OR anxious OR mood OR “mental health” OR “psychological wellbeing” OR “mental wellbeing” OR “behavioral health” OR “mental illness”)

AND

Title Abstract Keyword (online NEAR/5 intervention*) OR (online NEAR/5 treatment) OR (digital NEAR/5 intervention*) OR (digital NEAR/5 treatment) OR (mobile NEAR/5 intervention*) OR (mobile NEAR/5 treatment) OR (smartphone NEAR/5 intervention*) OR (smartphone NEAR/5 treatment) OR (web-based NEAR/5 intervention*) OR (web-based NEAR/5 treatment) OR (internet NEAR/5 intervention*) OR (internet NEAR/5 treatment) OR (computer NEAR/5 intervention*) OR (computer NEAR/5 treatment) OR (cyber NEAR/5 intervention*) OR (cyber NEAR/5 treatment) OR (electronic NEAR/5 intervention*) OR (electronic NEAR/5 treatment) OR (mobile program*) OR mhealth OR ehealth OR mtherap* OR etherap* OR telehealth OR telemedicine OR “mobile app*”)

AND

Title Abstract Keyword (usability OR “user experience” OR evaluation* OR engagement OR interface OR satisfaction OR usage OR adoption OR acceptability OR qualitative OR user perspective* OR barrier* OR interview* OR focus group*)
