# Supplementary material for: Barriers to and Facilitators of User Engagement With Digital Mental Health Interventions: Systematic Review
Source: J Med Internet Res. 2021 Mar 24;23(3):e24387. doi: 10.2196/24387 (PMC8074985; doi:10.2196/24387)
Supplement: Multimedia Appendix 3 [file jmir_v23i3e24387_app3.docx]

# Quality Assessment of Included Studies

| **Authors & Year** | **Question and**  **study design** | **Selection of**  **participants** | **Method of data**  **collection** | **Methods of data**  **analysis** |
| --- | --- | --- | --- | --- |
| Nicholas et al. 2010 [83] | Yes | Yes | Yes | Yes |
| Meglic et al. 2010 [127] | Yes | Yes | Yes | Yes |
| Postel et al. 2010 [150] | Yes | Yes | Yes | Yes |
| Klein & Cook 2010 [177] | Yes | Yes | Yes | Yes |
| Gulec et al. 2011 [41] | Yes | Yes | Yes | No |
| Saurman et al. 2011 [234] | Yes | Yes | Yes | Yes |
| Proudfoot et al. 2012 [52] | Yes | Yes | Yes | Yes |
| Doherty, Coyle & Sharry 2012 [51] | Yes | Yes | Yes | Yes (quantitative data)/No (qualitative data) |
| Donkin & Glozier 2012 [95] | Yes | Yes | Yes | Yes |
| Pagliari et al. 2012 [30] | Yes | No | Yes | Yes |
| Burda et al. 2012 [185] | Yes | Yes | Yes | Yes |
| Ye et al. 2012 [215] | Yes | Yes | Yes | Yes |
| Kaczmarek et al. 2013 [96] | Yes | Yes | Yes | Yes |
| Deen, Fortney & Schroeder 2013 [105] | Yes | Yes | Yes | Yes |
| Casey et al. 2013 [146] | Yes | Yes | Yes | Yes |
| Wilhelmsen et al. 2013 [14] | Yes | Yes | Yes | Yes |
| Crisp & Griffiths 2014 [53] | Yes | Yes | Yes | Yes |
| AL-Asadi, Klein & Meyer  2014 [97] | Yes | Yes | Yes | Yes |
| Brachel et al. 2014 [98] | Yes | Yes | Yes | Yes |
| Farrer et al. 2014 [99] | Yes | Yes | Yes | Yes |
| Habibović et al. 2014 [100] | Yes | Yes | Yes | Yes |
| Giroux et al. 2014 [179] | Yes | Yes | Yes | Yes |
| Gonzales, Anglin & Glik 2014 [180] | Yes | Yes | Yes | Yes |
| Boggs et al. 2014 [211] | Yes | Yes | Yes | Yes |
| Kasckow et al. 2014 [239] | Yes | Yes | Yes | Yes |
| Adler et al. 2014 [42] | Yes | Yes | Yes | No |
| Harjumaa et al. 2015 [44] | Yes | Yes | Yes | No |
| Grubbs et al. 2015 [101] | Yes | Yes | Yes | Yes |
| Forchuk et al. 2015 [128] | Yes | Yes | Yes | Yes |
| Lal et al. 2015 [131] | Yes | Yes | Yes | Yes |
| Woolderink et al. 2015 [132] | Yes | Yes | Yes | Yes |
| Bengtsson, Nordin & Calbring  2015 [135] | Yes | Yes | Yes | Yes |
| Ranney et al. 2015 [145] | Yes | Yes | Yes | Yes |
| Kenny, Dooley & Fitzgerald 2015 [166] | Yes | Yes | Yes | Yes |
| Saurman, Kirby & Lyle  2015 [236] | Yes | Yes | Yes | Yes |
| Baumel 2015 [235] | Yes | Yes | Yes | Yes |
| Schuster, Drennan & Lings  2015 [238] | Yes | Yes | Yes | Yes |
| Crosier et al. 2016 [62] | Yes | Yes | Yes | Yes |
| Kenny, Dooley & Fitzgerald  2016 [63] | Yes | Yes | Yes | Yes |
| Mattila et al. 2016 [64] | Yes | Yes | Yes | Yes |
| Pavliscsak et al. 2016 [69] | Yes | Yes | Yes | Yes |
| Forchuk et al. 2016 [84] | Yes | Yes | Yes | Yes |
| Nitsch et al. 2016 [85] | Yes | Yes | Yes | Yes |
| Fonseca, Gorayeb & Canavarro 2016 [106] | Yes | Yes | Yes | Yes |
| Newman, Bidargaddi & Schrader 2016 [118] | Yes | Yes | Yes | Yes |
| Chęć et al. 2016 [120] | Yes | Yes | Yes | Yes |
| Moessner et al. 2016 [31] | Yes | No | Yes | Yes |
| Wallin, Mattsson & Olsson  2016 [130] | Yes | Yes | Yes | Yes |
| Laurie & Blandford 2016 [133] | Yes | Yes | Yes | Yes |
| Anderson et al. 2016 [167] | Yes | Yes | Yes | Yes |
| Povey et al. 2016 [170] | Yes | Yes | Yes | Yes |
| Murnane et al. 2016 [173] | Yes | Yes | Yes | Yes |
| Lord et al. 2016 [174] | Yes | Yes | Yes | Yes |
| Huerta-Ramos et al. 2016 [178] | Yes | Yes | Yes | Yes |
| Ho et al. 2016 [197] | Yes | Yes | Yes | Yes |
| Kenny, Dooley & Fitzgerald  2016 [32] | Yes | No | Yes | Yes |
| Wentzel et al. 2016 [33] | Yes | No | Yes | No |
| Whealin et al. 2016 [187] | Yes | Yes | Yes | Yes |
| Aardoom et al. 2016 [195] | Yes | Yes | Yes | Yes |
| Clarke et al. 2016 [199] | Yes | Yes | Yes | Yes |
| Muuraiskangas et al. 2016 [200] | Yes | Yes | Yes | Yes |
| Schlosser et al. 2016 [34] | Yes | No | Yes | Yes |
| Pierce, Twohig & Levin 2016 [212] | Yes | Yes | Yes | Yes |
| Puszka et al. 2016 [35] | Yes | No | Yes | Yes |
| Koppe, van de Mortel & Ahern  2016 [216] | Yes | Yes | Yes | Yes |
| Bohleber et al. 2016 [217] | Yes | Yes | Yes | Yes |
| Naslund, Aschbrenner & Bartels  2016 [218] | Yes | Yes | Yes | Yes |
| Rickard et al. 2016 [45] | Yes | Yes | Yes | No |
| Townsend et al. 2016 [224] | Yes | Yes | Yes | Yes |
| Muuraiskangas et al. 2016 [225] | Yes | Yes | Yes | Yes |
| East, Havard, & Hastings 2016 [111] | Yes | Yes | Yes | Yes |
| Baumel & Schueller 2016 [232] | Yes | Yes | Yes | Yes |
| Simons et al. 2016 [246] | Yes | Yes | Yes | Yes |
| Espinosa et al. 2016 [198] | Yes | Yes | Yes | Yes |
| Beatty et al. 2017 [65] | Yes | Yes | Yes | Yes |
| Kannisto et al. 2017 [66] | Yes | Yes | Yes | Yes |
| Watson et al. 2017 [74] | Yes | Yes | Yes | Yes |
| Crooks et al. 2017 [78] | Yes | Yes | Yes | Yes |
| Heiniger et al. 2017 [86] | Yes | Yes | Yes | Yes |
| Gould et al. 2017 [102] | Yes | Yes | Yes | Yes |
| Adkins et al. 2017 [108] | Yes | Yes | Yes | Yes |
| Wozney et al. 2017 [129] | Yes | Yes | Yes | Yes |
| Rodriguez-Paras & Sasangohar  2017 [46] | Yes | Yes | Yes | No |
| Chan, West & Glozier  2017 [155] | Yes | Yes | Yes | Yes |
| Dodd et al. 2017 [156] | Yes | Yes | Yes | Yes |
| Powell, Parker & Harpin  2017 [157] | Yes | Yes | Yes | Yes |
| Stiles-Shields et al. 2017 [168] | Yes | Yes | Yes | Yes |
| Henshall et al. 2017 [172] | Yes | Yes | Yes | Yes |
| Huerta-Ramos et al. 2017 [113] | Yes | Yes | Yes | Yes |
| Lattie et al. 2017 [181] | Yes | Yes | Yes | Yes |
| Berry, Bucci & Lobban 2017 [186] | Yes | Yes | Yes | Yes |
| Nicholas et al. 2017 [188] | Yes | Yes | Yes | Yes |
| Saunders et al. 2017 [205] | Yes | Yes | Yes | Yes |
| Shrier & Spalding 2017 [206] | Yes | Yes | Yes | Yes |
| Bennett-Levy et al. 2017 [36] | Yes | No | Yes | Yes |
| Reger et al. 2017 [220] | Yes | Yes | Yes | Yes |
| Whealin et al. 2017 [221] | Yes | Yes | Yes | Yes |
| Nicholas et al. 2017 [229] | Yes | Yes | Yes | Yes |
| Melton et al. 2017 [47] | Yes | Yes | Yes | No |
| Vereenooghe, Gega & Langdon  2017 [240] | Yes | Yes | Yes | Yes |
| Possemato et al. 2017 [242] | Yes | Yes | Yes | Yes |
| Deady, M; Peters, D; Lang, H; Calvo, R; Glozier, N; Christensen, H; Harvey, S B;  2017 [37] | Yes | No | Yes | Yes |
| Apolinario-Hagen, Jennifer; Vehreschild, Viktor; Alkoudmani, Ramez M;  2017 [176] | Yes | Yes | Yes | Yes |
| Carolan, S; Harris, P R; Greenwood, K; Cavanagh, K;  2017 [196] | Yes | Yes | Yes | Yes |
| Abel et al.  2018 [67] | Yes | Yes | Yes | Yes |
| Graham et al. 2018 [68] | Yes | Yes | Yes | Yes |
| Gunn et al. 2018 [54] | Yes | Yes | Yes | Yes |
| Mackesy-Amiti & Boodrama 2018  2018 [55] | Yes | Yes | Yes | Yes |
| Mikolasek, Michael; Witt, Claudia M; Barth, Juergen;  2018 [56] | Yes | Yes | Yes | Yes |
| Schneider et al. 2018 [70] | Yes | Yes | Yes | Yes |
| March et al. 2018 [72] | Yes | Yes | Yes | Yes |
| Chudy-Onwugaje et al. 2018 [73] | Yes | Yes | Yes | Yes |
| Stevens et al. 2018 [75] | Yes | Yes | Yes | Yes |
| Mitchell et al. 2018 [80] | Yes | Yes | Yes | Yes |
| Arjadi, Nauta & Bockting 2018 [38] | Yes | No | Yes | Yes |
| Toscos et al. 2018 [81] | Yes | Yes | Yes | Yes |
| Meyer et al. 2018 [87] | Yes | Yes | Yes | Yes |
| Feijt et al. 2018 [103] | Yes | Yes | Yes | Yes |
| O'dea et al. 2018 [107] | Yes | Yes | Yes | Yes |
| Diez-Canseco et al. 2018 [115] | Yes | Yes | Yes | Yes |
| Thorsen et al. 2018 [119] | Yes | Yes | Yes | Yes |
| Mitchell et al. 2018 [134] | Yes | Yes | Yes | Yes |
| Walsh et al. 2018 [136] | Yes | Yes | Yes | Yes |
| Carolan & De Visser 2018 [140] | Yes | Yes | Yes | Yes |
| Lal, Nguyen & Theriault 2018  [143] | Yes | Yes | Yes | Yes |
| Deb et al. 2018 [147] | Yes | Yes | Yes | Yes |
| Chadi et al. 2018 [148] | Yes | Yes | Yes | Yes |
| Kubo et al. 2018 [149] | Yes | Yes | Yes | Yes |
| Ashford et al. 2018 [151] | Yes | Yes | Yes | Yes |
| Wallin et al. 2018 [153] | Yes | Yes | Yes | Yes |
| Schuster et al. 2018 [154] | Yes | Yes | Yes | Yes |
| Lundgren et al. 2018 [158] | Yes | Yes | Yes | Yes |
| Huis et al. 2018 [159] | Yes | Yes | Yes | Yes |
| Schuster et al. 2018 [161] | Yes | Yes | Yes | Yes |
| Similä et al. 2018 [162] | Yes | Yes | Yes | Yes |
| Wachtler et al. 2018 [163] | Yes | Yes | Yes | Yes |
| Miatello et al. 2018 [182] | Yes | Yes | Yes | Yes |
| Fortuna et al. 2018 [189] | Yes | Yes | Yes | Yes |
| Pung, Fletcher & Gunn  2018 [190] | Yes | Yes | Yes | Yes |
| Switsers et al. 2018 [114] | Yes | Yes | Yes | Yes |
| Schroeder et al. 2018 [201] | Yes | Yes | Yes | Yes |
| Terp et al. 2018 [203] | Yes | Yes | Yes | Yes |
| Williams et al. 2018 [204] | Yes | Yes | Yes | Yes |
| Cernvall et al. 2018 [207] | Yes | Yes | Yes | Yes |
| Kinner et al. 2018 [222] | Yes | Yes | Yes | Yes |
| Connolly et al. 2018 [226] | Yes | Yes | Yes | Yes |
| Krog et al. 2018 [230] | Yes | Yes | Yes | Yes |
| Apolinario-Hagen et al. 2018 [241] | Yes | Yes | Yes | Yes |
| Richards et al. 2018 [244] | Yes | Yes | Yes | Yes |
| Peters et al. 2018 [248] | Yes | Yes | Yes | Yes |
| Drozd et al. 2018 [247] | Yes | Yes | Yes | Yes |
| Caplan, Sosa & Reyna 2018 [175] | Yes | Yes | Yes | Yes |
| de Almeida et al. 2018 [48] | Yes | Yes | Yes | No |
| Stallman & Kavanagh  2018 [39] | Yes | No | Yes | Yes |
| Connolly et al. 2018 [226] | Yes | Yes | Yes | Yes |
| Achtyes et al. 2019 [57] | Yes | Yes | Yes | Yes |
| Krause et al. 2019 [58] | Yes | Yes | Yes | Yes |
| La Porte et al. 2019 [59] | Yes | Yes | Yes | Yes |
| Mira et al. 2019 [60] | Yes | Yes | Yes | Yes |
| Smail-Crevier et al. 2019 [61] | Yes | Yes | Yes | Yes |
| Ervasti et al. 2019 [77] | Yes | Yes | Yes | Yes |
| Eisner et al. 2019 [79] | Yes | Yes | Yes | Yes |
| Burchert et al. 2019 [88] | Yes | Yes | Yes | Yes |
| Görges et al. 2019 [89] | Yes | Yes | Yes | Yes |
| Kerr et al. 2019 [90] | Yes | Yes | Yes | Yes |
| Sayal et al. 2019 [91] | Yes | Yes | Yes | Yes |
| Schuster et al. 2019 [92] | Yes | Yes | Yes | Yes |
| Simblett et al. 2019 [93] | Yes | Yes | Yes | Yes |
| Berry, Lobban & Bucci  2019 [104] | Yes | Yes | Yes | Yes |
| Gindidis, Stewart & Roodenburg  2019 [116] | Yes | Yes | Yes | Yes |
| Bucci et al. 2019 [122] | Yes | Yes | Yes | Yes |
| Allan et al. 2019 [117] | Yes | Yes | Yes | Yes |
| Jordan & Shearer 2019 [121] | Yes | Yes | Yes | Yes |
| Urech et al. 2019 [137] | Yes | Yes | Yes | Yes |
| Anastasiadou et al. 2019 [138] | Yes | Yes | Yes | Yes |
| Saberi et al. 2019 [139] | Yes | Yes | Yes | Yes |
| Ashwick et al. 2019 [141] | Yes | Yes | Yes | Yes |
| Jonathan et al. 2019 [142] | Yes | Yes | Yes | Yes |
| Pretorius et al. 2019 [144] | Yes | Yes | Yes | Yes |
| Chen et al. 2019 [152] | Yes | Yes | Yes | Yes |
| Hamblen et al. 2019 [160] | Yes | Yes | Yes | Yes |
| Lipschitz et al. 2019 [164] | Yes | Yes | Yes | Yes |
| Hartmann et al. 2019 [165] | Yes | Yes | Yes | Yes |
| Gonsalves et al. 2019 [169] | Yes | Yes | Yes | Yes |
| Hunter-Jones et al. 2019 [171] | Yes | Yes | Yes | Yes |
| Almeida et al. 2019 [49] | Yes | Yes | Yes | No |
| O'Brien et al. 2019 [183] | Yes | Yes | Yes | Yes |
| Sreejith & Menon 2019 [184] | Yes | Yes | Yes | Yes |
| Thach 2019 [50] | Yes | Yes | Yes | No |
| Carr et al. 2019 [191] | Yes | Yes | Yes | Yes |
| Jarvis, Chipps & Padmanabhanunni 2019 [192] | Yes | Yes | Yes | Yes |
| Klein et al. 2019 [193] | Yes | Yes | Yes | Yes |
| Kim et al. 2019 [202] | Yes | Yes | Yes | Yes |
| Edbrooke-Childs et al. 2019 [208] | Yes | Yes | Yes | Yes |
| Garrido et al. 2019 [210] | Yes | Yes | Yes | Yes |
| Michalak et al. 2019 [223] | Yes | Yes | Yes | Yes |
| Brandt et al. 2019 [227] | Yes | Yes | Yes | Yes |
| Nicholas et al. 2019 [237] | Yes | Yes | Yes | Yes |
| Lemey et al. 2019 [231] | Yes | Yes | Yes | Yes |
| Navarro et al. 2019 [233] | Yes | Yes | Yes | Yes |
| Batterham et al. 2019 [43] | Yes | Yes | Yes | No |
| Schulze et al. 2019 [245] | Yes | Yes | Yes | Yes |
| Apolinario-Hagen et al. 2019 [109] | Yes | Yes | Yes | Yes |
| Clough et al. 2019 [40] | Yes | No | Yes | Yes |
| Huberty et al. 2019 [82] | Yes | Yes | Yes | Yes |
| Kemmeren et al. 2019 [76] | Yes | Yes | Yes | Yes |
| Lim et al. 2019 [94] | Yes | Yes | Yes | Yes |
| Pruitt et al. 2019 [71] | Yes | Yes | Yes | Yes |
